# Coaching leadership: leaders' and followers' perception assessment questionnaires in nursing

**DOI:** 10.1590/S1679-45082014AO2888

**Published:** 2014

**Authors:** Maria Lúcia Alves Pereira Cardoso, Laís Helena Ramos, Maria D'Innocenzo

**Affiliations:** 1 Escola Paulista de Enfermagem, Universidade Federal de São Paulo, São Paulo, SP, Brazil.

**Keywords:** Leadership, Nurse's role, Professional practice management, Personnel administration, hospital

## Abstract

**Objective:**

To describe the development, content analysis, and reliability of two questionnaires to assess the perception of nurse leaders, nurse technicians, and licensed practical nurses – coached in the practice of leadership and the relation with the dimensions of the coaching process.

**Methods:**

This was a methodological study with a quantitative and qualitative approach, which had the goal of instrumentation in reference to the construction and validation of measuring instruments. The instrument proposition design was based on the literature on leadership, coaching, and assessment of psychometric properties, subjected to content validation as to clarity, relevance, and applicability in order to validate the propositions through the consensus of judges, using the Delphi technique, in 2010. The final version of the questionnaires was administered to 279 nurses and 608 nurse technicians and licensed practical nurses, at two university hospitals and two private hospitals.

**Results:**

The Cronbach's alpha value with all items of the self-perception instrument was very high (0.911). The team members' instrument of perception showed that for all determinants and for each dimension of the coaching process, Cronbach's overall alpha value (0.952) was considered quite high, pointing to a very strong consistency of the scale. Confirmatory analysis showed that the models were well adjusted.

**Conclusion:**

From the statistical validation we compared the possibility of reusing the questionnaires for other study samples, because there was evidence of reliability and applicability.

## RESUMO

**Objetivo:**

Apresentar o desenvolvimento, a análise de conteúdo e a confiabilidade de dois questionários para avaliar a percepção de enfermeiros-líderes, técnicos e auxiliares de enfermagem – liderados sobre a prática da liderança e a relação com as dimensões do processo *coaching*.

**Métodos:**

trata-se de um estudo metodológico, de abordagem quanti-qualitativa, que teve como meta a instrumentação que se refere à construção e à validação de instrumentos de medida. A concepção das proposições dos instrumentos foi fundamentada na literatura sobre liderança, *coaching* e aferição das propriedades psicométricas, submetida à validação de conteúdo quanto a clareza, relevância e aplicabilidade, com o objetivo de validar as proposições por meio do consenso de juízes, utilizando a técnica Delphi, em 2010. A versão final dos questionários foi aplicada a 279 enfermeiros e 608 técnicos e auxiliares de enfermagem em dois hospitais universitários e dois hospitais privados.

**Resultados:**

O valor do alfa de Cronbach com todos os itens do instrumento de autopercepção foi muito alto (0,911). O instrumento de percepção dos liderados demonstrou que, tanto para todos os determinantes como para cada dimensão do processo *coaching*, o valor de alfa de Cronbach total (0,952) foi considerado bastante alto, apontando fortíssima consistência da escala. A análise confirmatória demonstrou que os modelos estavam bem ajustados.

**Conclusão:**

Pela validação estatística, comparou-se a possibilidade de reutilização dos questionários para outras amostras de estudo, pois houve evidências de confiabilidade e aplicabilidade.

## INTRODUCTION

Healthcare organizations indicate pathways linked to variables in reference to the threats and opportunities in the current market relationships. These variables are represented by social, educational, legal, technological, cultural, political and other inputs highly unpredictable in any environment. Hospitals, as a segment that fits into this scenario, should have personnel management in which leaders act in a dynamic, orchestrated, and interactive manner, carefully observing transformations of the external environment in order to bring about changes in the internal environment.

Leadership is an important factor that generates and applies energy in people, affording them direction and synchronizing their efforts. In fact, it represents the fundamental indicator of a company's potential, in contrast with financial results that only indicate where the company has been. Strong leadership makes a good company even stronger, in the same way that weak leadership reduces its potential and with time, destroys it.^(1)^

Hersey and Blanchard^(2)^ defined leadership as “the process of influencing the activities of an individual or a group in efforts toward goal achievement in a given situation.” For these authors, the definition is not connected with the type of organization; when an individual seeks to influence the behavior of another individual or of a group, relative to their activities within a company, school, or hospital, it can be said that they exercised leadership.

Within this approach, organizations need to transform themselves into organizational learning systems, in which team work is encouraged and people can acquire autonomy and self-actualization. Without this, they feel tied down and need to be freed, led, and encouraged. Coaching becomes an indispensable tool for selfcorrection of the behavior and of learning within the organization, and the coaches become elements necessary for its implementation.

Krausz^(3)^ has defined coaching as a process that contributes towards transformation of people and groups; with this they reflect on their world view, their values and beliefs; deepen their knowledge; incorporate new abilities and skills; and expand their readiness to act in a consistent and effective manner. For this reason, it is a constructive mode of provocation, of challenge and stimulation for ongoing development and learning.

The coaching process requires an extended organizational preparation, as each leader must be prepared and developed. Thus, coaching proposes a cultural change towards decentralization and participation, which requires a new mentality within organizations. The development of necessary skills for leaders is the objective.

In Brazil, the practice of coaching is relatively new, and there are few academic papers that refer to it, especially in the area of health and nursing. Recently, this practice was applied to evaluate the leadership given by nurses, proving a managerial strategy in personnel development.^(4)^

For the command of a few skills associated with leadership, we highlight the skills a nurse leader needs: to communicate; to give and receive feedback; to delegate power and exert influence; and to support the team so that the organizational results are reached – all these are dimensions of the coaching process.^(5)^

The development of leaders is the strategic and instrumental revitalization for providing capacities and perspective to managers, so that they are transformed into leaders-coaches of their followers-coachees, in search of concrete results and ongoing performance improvement. However, there is no study that evaluates the perception of nurses, nurse technicians, and licensed practical nurses (LPN) regarding leadership, considering the dimensions of the coaching process.

This study used investigative evaluation, with the objective of analyzing the perception of nurses, nurse technicians, and LPNs as to the exercise of leadership, considering the dimensions of the coaching process, hoping that the results help in producing specific knowledge and in constructing two quantification instruments for leadership competence in nursing.

## OBJECTIVE

To present the development, content analysis, and reliability of two questionnaires for the assessment of the perception of nurse leaders, technicians, and LPNs who are followers as to the practice of leadership and its relation with the dimensions of the coaching process.

## METHODS

The methodological procedures proposed for carrying out this investigation are divided into two stages. In the first, the dimensions of coaching leadership were analyzed with a premise related to its determinants, a premise that was constituted from a consensus on content of the instruments utilized by the judges in this evaluation, using the Delphi technique. The second encompassed outlining the research, with the study field in four large not-for-profit general hospitals located in the city of São Paulo (SP): two of them characterized as university hospitals, and two as private hospitals, during the period from June to December 2011. The leadership practice was analyzed, according to the perception of nurses, nurse technicians, and LPNs.

This is a methodological study, with a quantitative and qualitative approach that had as goal the instrumentation in reference to the construction and validation of measurement instruments, that is, that seeks “the construction and validation of formal quantitative instruments with research or clinical purposes.”^(6)^ As a theoretical-methodological reference for the adaptation and creation of the instruments, Pasquali's assumptions were used.^(7)^ According to them, in general, the assessment instruments should be clear, comprehensible, easily applied, and with the possibility of use in various contexts.

### Ethical aspects

The project was approved by the Research Ethics Committee of the *Universidade Federal de São Paulo*, under no. 1,405/2009. The Informed Consent Form (ICF) was previously signed by the participating judges and subjects.

### Development of the instruments

The research instruments were created and based on the experience of the investigator, and the propositions were based on literature about leadership in the light of the theoretical reference of Situational Leadership, by Hersey and Blanchard,^(2)^ amply utilized in Nursing studies, as well as on an extensive bibliographic search on coaching.

Questionnaires were used and one was directed to nurses (leaders) and entitled Questionnaire on Self-Perception of Nurses of Exercise of Leadership (QUAPEEL, abbreviation in Portuguese – Appendix 1). The other questionnaire was for nurse technicians and LPNs (followers), called Questionnaire on Perception of Nurse Technicians and LPNs of Exercise of Leadership (QUEPTAEEL, abbreviation in Portuguese – Appendix 2). Both questionnaires contain structured questions and are composed of three parts: the first is formed by sociodemographic data of the subjects; the second, with open- and closed-ended questions relative to the subjects' knowledge about the theme of leadership; and the third with questions relative to the abilities and attitudes of the leaders and followers in the practice of coaching leadership. The latter is composed by scalar measurement instrument.

The scale is a device destined to attribute a numerical score to people, and is frequently incorporated into a questionnaire.^(6)^ In this study, Likert's scale was used, with six variation fields; the participants were requested to indicate de degree of agreement and disagreement with the statements.

At the end of each instrument, a specific closed question was also included for the general classification of the instruments built. The intent of the question was to evaluate its applicability in healthcare organziations, specifically within the hospital context. The following measurement was used: fully applicable, applicable, relatively applicable, and not applicable.

### Validity of the content

The validity of the content represents the content or the domain of a given construct. The universe of the content provides the bases for the formulation of the criteria that will represent it. Initially, it is an issue of defining the concept and identifying the component dimensions of the concept, and then the criteria are formulated. When the investigator completes this task, he/she submits the tool, or in this case, the instrument to a group of judges considered specialists in this concept.^(8)^

To verify the validity of the content, the instruments were submitted to the evaluation of judges with knowledge and experience in the area who fulfilled at least two of the following criteria: management, teaching, research, and consulting in the area of nursing and leadership of people, were members of the Brazilian Society of Nursing Management (SOBRAGEN), and had publications on the theme studied. From this group of 24 judges, 5 were administrators, engineers, and psychologists specialized in staff management and coaching and 19 were nurse managers and nursing leaders.

After informal contact, the judges received a letter of invitation and agreement to participate in the study, accompanied by a copy of the instruments and instructions for administrators, and by the number of points of the scores and content validation questionnaires.

As to the clarity, the judges used the evaluation made by the instruments as parameters, considering when the proposition was objective, comprehensible, and used unambiguous expressions; as to relevance, if it was significant to know the beliefs of the research participants, the content and pertinence of the statements in each one of the dimensions, as well as the need to include or exclude a proposition. The scalar interval was also evaluated as to being appropriate, excessive, or insufficient.

In order to achieve consensus, a minimal percentage of 75% agreement among the judges was established. For this, two necessary cycles were performed to reach the minimum values established.

After each Delphi technique phase, the data obtained were inserted by the investigator into a Microsoft Excel^®^ spreadsheet containing the code of the instruments (increasing ordinal numbers) and the degree of agreement among the judges was verified. All answers were compiled into a list, with the opinion of the responders to each element, so that all the modifications made were declared, as per the judgment of the proponents.

The resulting data from consensus among the judges were analyzed under orientation of a biostatistician and the following methods were used: validation of the instrument by specialists and evaluation of internal consistency. It was only after content validation of the research instruments by the experts and adjustments necessary for their suitability that the next phase of the investigation occurred: the pre-test.

### Application of research instruments: pre-test

In this phase of the study, it was possible to evidence if the requirements related to the vocabulary and clarity of the questions were adequate, assuring that the study variables were measured. The few suggestions given by nurses and nurse technicians as to suitability of the instruments were received by the researcher since they were considered relevant, favoring the visualization and formatting of the questionnaire.

The final version of the questionnaires was applied to 279 nurses and 608 nurse technicians and LPNs working in the medical/surgical clinic admission units, adult intensive care units, adult emergency rooms, operating rooms, and central sterilization and supplies departments who met the eligibility criteria.

## RESULTS

The two questionnaires (Appendices 1 and 2) were prepared in three parts. The third part had 20 statements, called determinants of the coaching process dimensions; for each dimension, there were four situations based on the skills and attitudes of the nurse leader, graduated in an increasing manner as to the practice of leadership, with scores from 5 to 1. The perceptions of the leader and followers consisted of the analysis of the dimensions relative to each of the 20 determinants and each option of the situation that best represented the exercise of leadership.

Four dimensions of the coaching process were evaluated: communication, giving and receiving feedback, delegating power and exerting influence, and support the team to attain the organizational results. The assessment of content validity of question from part three of the instruments QUAPEEL and QUEPTAEEL, and the respective determinants of each group of judges who supported the modifications in order to be suitable to the reality of nursing leadership practice.

It is noted that the agreement among judges in the first cycle varied from 61.9% to 95.2%, with a mean percentage of 78.8%. As to the determinants proposed, if clear and relevant, the option was “agreed”; if clear and not relevant, the option was “disagreed”; and if the question was relevant but not clear, the option was “modify”. In this case, the experts proposed suggestions for the modifications of the conceptual definitions.

In the second cycle of the Delphi technique, the determinants of analysis of self-perception of nurses as to the exercise of leadership were scored by the judges, between the minimal percentage of 81.2% and maximal of 93.7%. Of the six determinants of skills and attitudes shown by nurse leaders of the QUAPEEL instrument, two had to be adapted, and in QUEPTAEEL, three ended with adjustments as to sentence writing and correction of the language, as well as simple suggestions to improve the text. None of the determinants proposed was excluded or considered not pertinent to the theme. For the latter, the agreement among the judges varied between 56.3 and 93.8%, with a mean percentage of 76.4%.

In the opinion of the judges, there was reliability of the scales regarding the results of the Cronbach's alpha for QUAPEEL, which was low for the first cycle (0.466) and high for the second (0.698); for QUEPTAEEL, during the first cycle it was very high (0.821) and in the second cycle it was high (0.608). The psychometric scale was considered appropriate by 16 (76%) judges, and the applicability of the instruments fully applicable by 13 (68%), and applicable by 6 (32%) judges.

Tables 1 and 2 contain the results of the analysis of internal consistency of all determinants of the coaching process proposed for the questionnaires assessed by means of the Cronbach's alpha, considering a significance level of 5%. Both questionnaires pointed to a very strong consistency of the scale.

**Table 1:** Distribution of Cronbach's alpha for all the determinants of the coaching process relative to the nurses’ instrument – Questionnaire on Self-perception of Nurses in Exercising Leadership

Determinants of the coaching process	Correlation of the item with the total correlation	Alpha – if the item was deleted
4.1. I know how to listen to the followers	0.451	0.899
4.2. I can maintain interest in the followers regarding maintenance and continuation of the dialogue	0.487	0.898
4.3. I give orientations to the followers and counsel them, meeting their professional needs	0.422	0.899
4.4. I use verbal communication and pay attention to non-verbal communication in dialogue with followers	0.373	0.901
4.5. I contribute toward effective communication in work relations with the followers	0.416	0.900
4.6. I give orientations to the followers according to their individual needs demonstrating how the tasks should be done	0.580	0.895
4.7. I answer questions of the followers regarding their tasks	0.489	0.898
4.8. I recognize and value the followers for what they do or for how they behave	0.485	0.898
4.9. I give orientation to the followers showing a new path to follow when they do not correspond to the performance expected	0.533	0.897
4.10. I periodically accompany the performance of the followers	0.584	0.895
4.11. I encourage the practice of giving and receiving feedback with the followers	0.556	0.896
4.12. I exert influence over the followers in favor of conditions to expand their skills in the search for effective results	0.585	0.895
4.13. I share the decisions with the followers relative to their activities	0.551	0.896
4.14. I delegate activities to the followers, sharing responsibilities	0.530	0.897
4.15. I contribute towards the professional development of my followers	0.561	0.896
4.16. I help the followers when they are facing a professional difficulty	0.582	0.895
4.17. I value the opinion of the followers in order to modify a procedure or propose an operational change	0.537	0.897
4.18. I establish, along with each follower of my team, the goals to be reached	0.690	0.892
4.19. I periodically accompany each follower as to the results presented	0.646	0.893
4.20. I make an agreement as to the timeframe necessary for each follower to reach his/her goals	0.550	0.897
Alpha total		0.911

**Table 2:** Distribution of Cronbach's alpha for all determinants of the coaching process, relative to the instrument of nurse technicians and LPNs – Questionnaire on Perception of nurse technicians and LPNs in Exercising Leadership

Determinants of the coaching process	Correlation of the item with the total correlation	Alpha - if the item is deleted
4.1. My leader listens to me	0.694	0.950
4.2. I receive attention and the interest of the leader in keeping dialogue	0.722	0.950
4.3. I am given orientations and counseling from the leader when I need to meet my professional needs	0.730	0.950
4.4. I use verbal communication and pay attention to non-verbal communication in dialogue with my leader	0.545	0.952
4.5. I contribute towards effective communication in work relations with my leader	0.547	0.952
4.6. I receive orientation from my leader and demonstrations of how I should perform the tasks, according to my needs	0.641	0.951
4.7. I receive answers to my questions from my leader, when I have inquiries about my tasks	0.620	0.951
4.8. I am known and valued by the leader for what I do or for how I behave	0.741	0.949
4.9. I am oriented to follow a new path when I do not correspond to the performance expected	0.650	0.951
4.10. I am accompanied periodically in my performance	0.660	0.951
4.11. I receive from and give feedback to the leader	0.706	0.950
4.12. I am influenced by my leader, expanding my skills in search of effective results	0.785	0.949
4.13. My leader shares the decisions with me	0.750	0.949
4.14. I receive orientation from my leader to exert the activities and I perceive sharing of responsibilities	0.783	0.949
4.15. My leader contributes towards my development	0.802	0.948
4.16. My leader is at my disposal to help me when I am facing a professional difficulty	0.727	0.949
4.17. My leader values my opinion to modify a procedure or propose an operational change	0.712	0.950
4.18. My leader defines with me the goals to be reached	0.737	0.949
4.19. My leader periodically accompanies the results presented by me	0.755	0.949
4.20. I know the timeframe foreseen for the goals to be reached by me	0.551	0.952
Alpha total		0.952

In table 1, the value of Cronbach's alpha observed was very high (0.911) for QUAPEEL and in table 2, there was a very high value (0.952) for QUEPTAEEL. The Cronbach's alpha value varied from 0 to 1 and indicates greater consistency the closer it gets to 1. Thus, the confirmatory analysis demonstrated that the models were well adjusted.

From the data on table 3, it was noted that there was a statistically significant difference between the perception of the leaders as to all dimensions, with a mean of 84.4 (standard deviation – SD:8.8), and of the followers, with a mean of 81.4 (SD:12.5) – p value=0.001; among the dimensions, communications and perception of the leaders, with a mean of 21.43 (SD:2.15), and of the followers, with a mean of 22.2 (SD:2.5) – p value <0.001; and concerning giving and receiving feedback for the leaders, with a mean of 21.80 (SD:2.35), and followers, with a mean of 21.0 (SD:3.2) p value=0.008 by Student's t test.

**Table 3:** Distribution of the statistical analysis and p-value of Student's t test according to the comparison of perception of the dimensions of the coaching process among nurse leaders, nurse technicians, and LPNs from all hospitals

Determinants	n	Mean	Median	Standard deviation	Minimum	Maximum
Total score (leader)*	249	84.4	85	8.8	51	100
Total score (follower)*	249	81.4	84.2	12.5	39	100
Communication (leader)*	249	21.43	21	2.15	14	25
Communication (follower)*	249	22.2	23	2.5	8	25
Give and receive feedback (leader)**	249	21.8	22	2.35	14	25
Give and receive feedback (follower)**	249	21	22	3.2	9.5	25
Delegate power and exert influence (leader)***	249	20.99	21	2.75	12	25
Delegate power and exert influence (follower)***	249	20.3	21.2	3.8	6.5	25
Support the team in reaching the results (leader)****	249	20.19	21	3.38	6	25
Support the team in reaching the results (follower)****	249	21	3.8	5	25	20.15

* p value: 0.001;

** p value: 0.008;

*** p value: 0.165;

**** p value: 0.987.

In the opinion of the 279 nurses, the QUAPEEL instrument of self-perception of the exercise of leadership was considered fully applicable for 78 (28.0%), applicable for 178 (63.8%), and relatively applicable for 23 (8.2%) nurses. There was no option for the “not applicable” alternative, thus showing its applicability. Among the 606 (99.6%) responding followers, the QUEPTAEEL instrument was considered applicable for 358 (59.1%), fully applicable for 169 (27.9%), relatively applicable for 74 (12.2%), and only 5 (0.8%) nurse technicians and LPNs opted for the “not applicable” alternative.

## DISCUSSION

The objective of this study was to present the development and the content analysis of reliability of two questionnaires destined to assess the perception of nurse leaders, nurse technicians, and LPNs – coached on the practice of leadership and the relationship with the dimensions of the coaching process. The findings demonstrated that they are conceptually valid instruments, comprehensible to the nurses, nursing technicians, and LPNs, and consistent in the attributes they measure.

It is understood that the findings established a consensus of concept of the situations that represents skills and attitudes exerted by nurse leaders and their followers in the practice of coaching leadership, as per agreement and the suggestions stated by the judges, as it considers that they were pertinent to the content of each proposition.

There is positive evidence as to the reliability of the QUAPEEL and QUEPTAEEL instruments, since the questionnaires proved fully applicable and highlighted the fact that the agreement of representativeness of the determinants, in face of the universe of coaching leadership, was consistent and demonstrated solid content.

The validity of content has been defined as the degree to which the instrument has an appropriate set of items to represent a construct^(9)^ or, yet, the extension by means of which an instrument adequately represents the domain of interest in seeking to measure a given phenomenon.^(10)^

The initial instruments until the final version were carefully modified during the two phases. All the 20 determinants were treated with uniform importance, but it is believed that in practice, some determinants (such as those of the communication dimension) may have been considered more determinant to the nurse leader in exerting coaching leadership.

In this regard, the result confirms that communication is a crucial instrument in the leadership process, as it is a resource that allows the leader to come close to the followers with the intent of understanding the activities of each one, sharing ideas and visions, as well as to create interdependencies for the development of the work by means of teams.^(11)^

Considering the results presented, and bearing in mind the perception of nurse technicians and LPNs, one can affirm that there was consistency with the perceptions of the nurses. It was further noted that the followers have a positive view in declaring the practical applicability of the dimensions of the coaching process and in admitting that they acknowledged the leadership given by the nurse leader in day-to-day work.

## CONCLUSION

By means of statistical validation, the possibility of use of the QUAPEEL and QUEPTAEEL instruments with other study samples was considered, since there was evidence of reliability and applicability. Even though they were used at the level of evaluation of perception and opinion, the possibility of their use at levels of application and impact evaluation was clear, in agreement with the practice of coaching leadership in nursing.

The QUAPEEL and QUEPTAEEL instruments may be used in hospitals as a mapping strategy by nurse leaders and in identifying the effects of adopting practices that influence the coaching process. The considerations are suggestive, since in order to outline the effective performance of this practice, it would be necessary to experience the measurement model proposed.

Since this is one of the first academic papers, if not the first, to directly focus on the practice of leadership in nursing, considering the dimensions of the coaching process in Brazil, it is clear that much still needs to be investigated to a fuller extent in this regard.

## Appendix 1 Self-Perception Questionnairefor the Nurse in the Exercise of Leadership

[Fig f1]

## Appendix 2 Questionnaire on Perception of nurse technicians and LPNs in Exercising Leadership

[Fig f2]
